# Visualization of Forward Light Scatter in Opacified Intraocular Lenses and Straylight Assessment

**DOI:** 10.3390/diagnostics11081512

**Published:** 2021-08-22

**Authors:** Hyeck-Soo Son, Grzegorz Łabuz, Ramin Khoramnia, Timur M. Yildirim, Chul Young Choi, Michael C. Knorz, Gerd U. Auffarth

**Affiliations:** 1 The David J. Apple International Laboratory for Ocular Pathology and International Vision Correction Research Centre (IVCRC), Department of Ophthalmology, University of Heidelberg, 69117 Heidelberg, Germany; son.hyecksoo@gmail.com (H.-S.S.); grzegorz.labuz@med.uni-heidelberg.de (G.Ł.); ramin.khoramnia@med.uni-heidelberg.de (R.K.); timur.yildirim@med.uni-heidelberg.de (T.M.Y.); chulyoung.choi@gmail.com (C.Y.C.); 2 Department of Ophthalmology, Kangbuk Samsung Hospital, Sungkyunkwan University School of Medicine, Seoul 03181, Korea; 3 FreeVis LASIK Center, Medical Faculty Mannheim of the University of Heidelberg, 68167 Mannheim, Germany; knorz@eyes.de

**Keywords:** ray propagation, imaging technique, intraocular lens, opacification, straylight

## Abstract

Background: Qualitative visualization of forward light scatter and quantitative straylight measurement of intraocular lenses (IOLs). Methods: We analyzed two calcified IOL-explants, the Euromaxx ALI313Y (Argonoptics GmbH) and the LS-312 MF30 (Oculentis BV), one IOL with artificially induced glistenings (PC-60AD, Hoya), and one control (CT Asphina 409MP, Carl Zeiss Meditec AG) free of any opacification. Analysis included light microscopy, qualitative light scatter visualization using ray propagation imaging technique, and quantitative straylight measurement using C-Quant (Oculus). Results: More light scattering effect—visible as increased light intensity outside the IOL’s main focus—was evident in all opacified IOLs than the control. The highest straylight levels were observed in the Euromaxx (289.71 deg^2^/sr), which showed extensive granular deposits throughout its optic, followed by the MF30 (78.58 deg^2^/sr), which only showed opacification in its center. The glistenings-IOL demonstrated numerous microvacuoles within the optic and had straylight levels of 22.6 deg^2^/sr, while the control showed the lowest straylight levels (1.7 deg^2^/sr). Conclusions: Ray propagation imaging technique allowed qualitative assessment of off-axis veils of light that result from increased forward light scattering. Straylight was increased in all opacified lenses compared to the clear control lens. The IOL opacifications are significant sources of glare.

## 1. Introduction

Intraocular lens (IOL) opacification is a rare but serious complication that can severely degrade quality of vision and even require an IOL explantation [1,2,3,4,5,6,7,8,9,10,11,12,13]. Generally, one can distinguish between two forms of IOL opacification depending on the IOL biomaterial [4,14,15]. On the one hand, IOLs made of hydrophobic acrylic material have the propensity to develop glistenings (small, fluid-filled microvacuoles) over time within the lens material, and these glistenings opacify the lens [16,17,18]. On the other hand, hydrophilic acrylic IOLs are associated with risks of developing calcification [4,13,14,15]. Apple and associates categorized this form of opacification into primary, secondary, and “pseudo”-calcifications depending on the cause and nature of the calcification [13].

Regardless of the opacification type, there are many reports showing that both calcification and glistenings may lead to a reduction in light transmission, loss of contrast sensitivity, and an increase in light scattering, ultimately deteriorating the patients’ quality of vision and life [4,14,15,19]. While the IOL manufacturers set out to improve and develop new materials to avoid these complications, the clinician nevertheless continues to be confronted with pseudophakic patients who complain of visual impairment, and ocular examination reveals that they have an opacified, older IOL model.

In a previous publication, we described a ray-imaging technique that allows one to visualize the course of light rays through a lens, and we demonstrated this with different IOL designs [20]. This technique allows a qualitative assessment of the incident light rays’ trajectory. We speculated that although we had used the method with new IOLs, it might also be applied to examining opacified IOLs. If one could visualize the forward light distribution and also quantify the scattering of light in explants, would this give a better understanding of the optical disturbance in the patient’s eye prior to explantation? To what extent might our in vitro results explain the patient’s in vivo experience? This was our aim in the present research: to analyze the light scatter effects of the opacified IOLs using the same experimental set-up and to compare their straylight levels to those of a clear control IOL.

## 2. Materials and Methods

### 2.1. Intraocular Lenses

The following opacified IOL-explants were studied: a hydrophilic acrylic Euromaxx ALI313Y (+23.5 D; Argonoptics GmbH, Haltern am See, Germany) and a hydrophilic acrylic LS-312 MF30 (+21.0 D; Oculentis B.V., Eerbeek, The Netherlands) IOL. These IOLs were donated to our laboratory for analysis by surgeons who explanted the IOLs due to severe opacification. Our methodology for handling such IOLs is described in an earlier paper by Tandogan et al. [12].

In addition to the explant specimen, a hydrophobic acrylic PC-60AD (+21.0 D; Hoya, Chromos, Singapore) IOL with laboratory-induced glistenings was included in our analysis. The glistenings were induced in vitro using the same, widely established method as described in previous studies [16,17,18].

As a control IOL, we chose a freshly unpacked and thus free of any opacification, CT Asphina 409MP (+21.0 D; Carl Zeiss Meditec AG, Berlin, Germany). This IOL is composed of hydrophilic acrylic material with a hydrophobic surface.

### 2.2. Morphological Analysis

To identify the morphology and pattern of opacification, gross microscopic images of the IOLs were taken using an Olympus BX50 light microscope and an Olympus C-7070 digital camera (Olympus Optical Co. Ltd., Tokyo, Japan). For the PC-60AD IOL, the images were taken with a different, EMZ-8TR Trinocular Zoom Stereo microscope (Meiji Techno, Saitama, Japan) to better visualize the whitish, opaque glistenings within the lens material.

For each IOL, one overview as well as two close-up images were taken from the opacified part of the optic to better visualize the distribution of the opacification.

### 2.3. Ray Propagation Imaging

**Figure 1:**
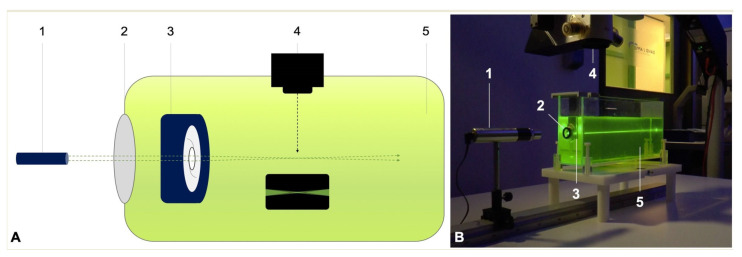
Experimental set-up for direct visualization of the light scattering effects. (**A**) Schematic illustration, (**B**) in vitro optical bench set-up. To elaborate, 1 = monochromatic green laser light source (532 nm); 2 = model cornea; 3 = intraocular lens holder; 4 = surgical microscope with an integrated digital camera; and 5 = water bath containing 0.01% fluorescein solution.

Light distribution of the IOLs was visualized using the same technique as we described previously (Figure 1) [20]. In an experimental set-up, the studied IOL was placed into an IOL holder submerged in a water bath (1 L) with fluorescein solution. A red-orange fluorescein solution (Alcon Laboratories Inc., Fort Worth, TX, USA) was used for injection with 100 mg/mL concentration of fluorescein loaded from a 5 mL glass vial. A monochromatic Gaussian light beam was then projected through a model cornea (*f* = 30 mm) and through the IOL under test.

We used a green laser light (532 nm) with a fixed power of 1 mW. The visualized light distribution was captured with a digital camera mounted on a surgical microscope (Leica Camera AG, Wetzlar, Germany) using 40x magnification. Following background-noise subtraction, the captured images were then converted to log images using custom-made software (Version R2021a, Image Processing Toolbox, Matlab; MathWorks, Inc., Natick, MA, USA). All images shared the same camera setting of 1/4 shutter speed and 400 ISO sensitivity with all automatic features switched off.

### 2.4. Straylight Measurements

The IOLs’ straylight levels were assessed using a modified straylight meter, the C-Quant (Oculus Optikgeräte GmbH, Wetzlar, Germany). This device measures the straylight levels at an effective 7° scatter angle [19]. While the C-Quant is generally used to assess the in vivo light scattering of the eye in a clinical setting, the modification allows measurement of in vitro straylight originating from the IOL itself, independent of the examiner’s eye. The principles of this modification have been described in previous studies and the protocol is well established [2,18,19]. This study followed the same protocol.

The C-Quant calculates the levels of straylight using the formula:

Log(s) = θ^2^ × PSF (θ) (deg^2^/sr)

where θ is the effective scatter angle and the point spread function (PSF) expresses the light intensity per steradian [21,22].

In order to dismiss any potential straylight level resulting from the optical setup itself, the straylight levels of the setup without the IOL under test was measured prior to measurements and later subtracted from the values measured with the IOLs in place. For each sample, we performed two independent measurements.

Additionally, straylight levels from the studied IOLs were compared to those of a 20-year old human crystalline lens and a 70-year old one, and a cataractous lens. These normative data were derived from the International Commission on Illumination standard [23,24].

## 3. Results

### 3.1. Morphological Analysis

**Figure 2:**
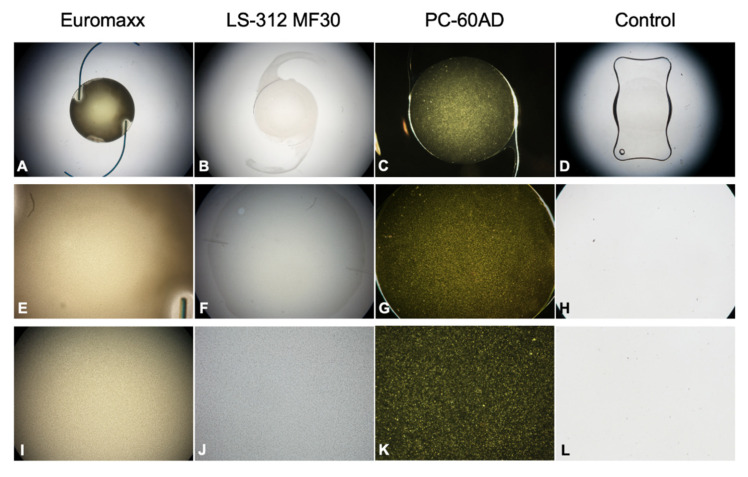
Gross microscopic images of the studied IOLs. (**A**–**D**) Overview images, (**E**–**H**) images taken with 4-fold magnification, (**I**–**L**) images taken with 40-fold magnification.

Figure 2 shows the gross microscopic images of the studied IOLs.

The Euromaxx IOL showed extensive calcification across its entire optic, only the haptic-optic junction area was spared (Figure 2A). Four-fold and 40-fold magnification revealed numerous fine, granular, densely packed crystalline-like deposits distributed evenly in the affected area (Figure 2E,I). While a similar pattern and density could also be observed in the LS-312 MF30 (Figure 2J), the calcification was limited to the central area of the optic (Figure 2B,F).

The PC-60AD IOL showed a large number of small, whitish glistenings (microvacuoles) in the central optic area (Figure 2C,G,K). The control IOL did not show any type of lens opacification (Figure 2D,H,L).

### 3.2. Ray Propagation Imaging

#### 3.2.1. Control IOL

**Figure 3:**
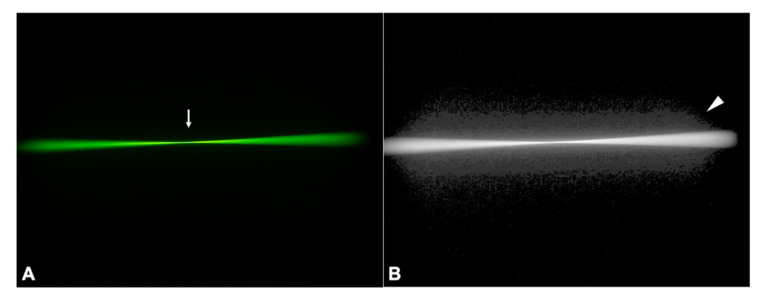
Ray propagation (**A**) and light scattering (**B**) of the control IOL. The arrow indicates the focal point of the studied IOL, while the arrowhead points to the scatter light made visible as background haze when the ray propagation image is converted to a log image.

In ray propagation imaging, the monofocal control IOL showed mostly lucent light rays that refracted to one focus (Figure 3A, arrow). Once converted to a log image, a cross-excitation of the medium surrounding the very bright light cone and fluorescein-particle scattering were made visible (Figure 3B, arrowhead).

#### 3.2.2. PC-60AD

**Figure 4:**
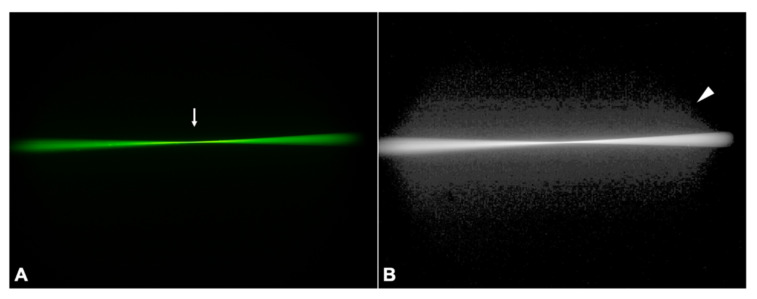
Ray propagation (**A**) and light scattering (**B**) of the glistenings-IOL. The arrow indicates its focal point, while the arrowhead points to its extent of scatter light.

While the monofocal PC-60AD also allocated its light energy to a single focal point (Figure 4A, arrow), its log image demonstrated more scatter light than the control IOL (Figure 4B, arrowhead).

#### 3.2.3. LS-312 MF30

**Figure 5:**
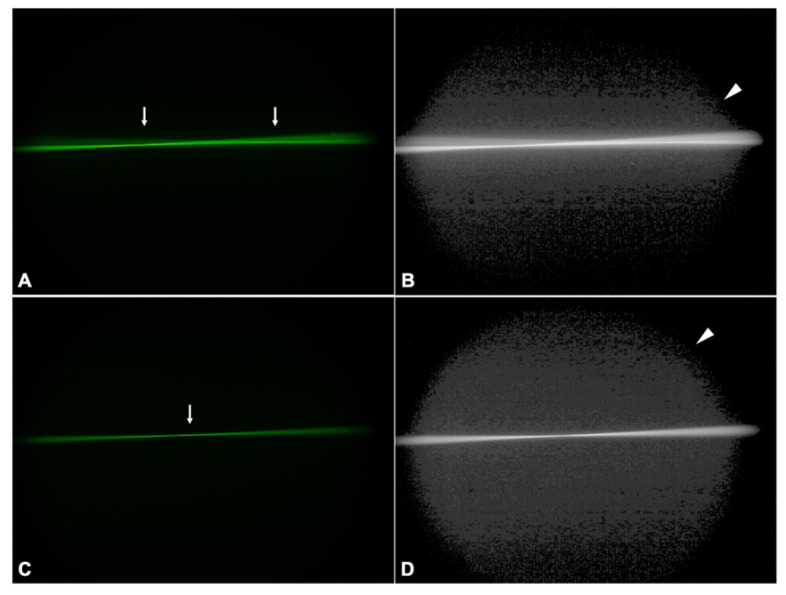
Ray propagation and light scattering of the calcified LS-312 MF30 (**A**,**B**) and Euromaxx (**C**,**D**) IOL-explants. The arrows indicate the focal point, while the arrowheads point to the scatter light.

Reflecting its bifocal nature, the LS-312 MF30 distributed incident light to two focal points (Figure 5A, arrows). The corresponding log image also unveiled a diffuse background haze that was most intense in the area directly adjacent to the light rays (Figure 5B, arrowhead), only to dissipate quickly in the image periphery.

#### 3.2.4. Euromaxx ALI313Y

The Euromaxx showed dim light rays that refracted to a single focal point (Figure 5C, arrow). The area of scattered light was the greatest compared to the other studied lenses, expanding perpendicularly to the optical axis even though the camera settings were the same for all IOLs (Figure 5D, arrowhead).

### 3.3. Straylight Measurements

**Figure 6:**
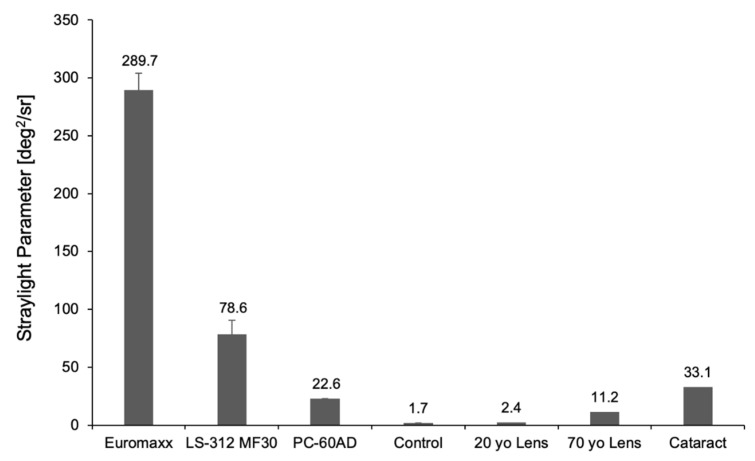
Straylight parameter of the studied IOLs measured by the C-Quant device. The results are compared to the normative values of a 20-year-old crystalline lens, 70-year-old crystalline lens, and a cataract lens [23,24].

Figure 6 illustrates the straylight of the studied IOLs compared to those of a 20-year-old, 70-year-old, and a cataract lens. The highest amount of straylight was observed in the Euromaxx, followed by the LS-312 MF30. The two IOL-explants’ straylight levels were much greater than those of a cataractous lens. While the straylight levels of the PC-60AD were lower than those of the IOL-explants or the cataract lens, it was considerably higher than those of the control IOL. The control IOL showed the lowest levels of straylight, lower than those of a 20-year-old lens.

## 4. Discussion

In this study, we implemented the imaging technique [20] to assess the ray propagation and forward light scatter of opacified IOLs and a clear IOL, and we found a good agreement between the qualitative (imaging technique) and quantitative (straylight) measurements. Previously, a similar experimental setup was utilized to understand the fundamental properties of IOLs [25,26,27]. Terwee et al. demonstrated the functional differences between diffractive and refractive multifocal IOLs by using monochromatic green light and United States Air Force target projections [25], while Eppig et al. used a different medium, namely ouzo, to demonstrate the halo effects of monofocal and multifocal lenses [27]. To our knowledge, this is the first study to use the experimental set-up to visualize the veil of light produced by opacified IOLs.

IOL calcification has been demonstrated to be associated with markedly increased straylight [2,7,19,26]. Straylight, which is defined as the perceptual quantity that corresponds to the functional light scatter in an eye, may be perceived by patients as hazy vision, reduced contrast sensitivity, or both [19,28,29,30,31]. The symptoms may be so severe that they even necessitate IOL explantation [4,6,14,15]. A recent review of 200 IOL-explants found that primary IOL calcification was by far the most common cause of explantation, accounting for 76.5% of all cases [6].

As IOL calcification is difficult to induce in a laboratory setting, calcified IOL-explants are commonly used for in vitro analysis. The two IOL-explants analyzed in the present study are composed of materials known to cause severe primary calcification [6,7,12]. Scanning electron microscopy and X-ray spectroscopy of explanted Euromaxx IOLs showed calcium and phosphate deposits densely distributed in a line parallel to the anterior and posterior surfaces of the IOLs [12]. The calcified Euromaxx IOLs also demonstrated a significant reduction in their optical quality, an effect that is attributable to the extensive calcification [12]. Calcified LS-312 MF30 explants have been associated with significantly increased straylight levels [7].

Our qualitative analysis of the calcified IOL-explants confirmed the findings of the previous studies. The dedicated imaging technique allowed visualization of not only the incident light rays that refracted to the focal points, but also the scattering effects of the opacification, which were visible as profoundly increased background haze surrounding the light rays. The impact was more noticeable in the Euromaxx IOL, which demonstrated densely distributed granular deposits across its entire optic, compared to the LS-312 MF30, which showed calcification that was limited to its central optic. In fact, such a central opacification is a pattern typically observed in secondary calcification, and it is therefore likely that the LS-312 MF30′s opacified optic is due to both primary and secondary calcification. As the explanting surgeon did not provide any details about any preceding ocular surgical interventions, however, it is not known to us if the LS-312 MF30 IOL was subject to any change in aqueous humor composition prior to explantation.

The fact that the incident light rays of the Euromaxx IOL were dimly illuminated even though all images were taken under the same camera setting, is suggestive of the loss of light transmission and visual quality impairment that the patient must have experienced before explantation. Although the bright area near the optical axis was observed in the ray-propagation images of all the studied IOLs, including the control lens, the peripheral image portion of the opacified IOLs was more affected by scattering effects. Given that calcium precipitates and glistening are large compared to the wavelength used, as opposed to fluorescein compounds, the increased intensity area observed in the log images may result from confounding large (Mie) [32] and small (Rayleigh) particle scattering [33].

The measured straylight parameter of both calcified IOLs was also in accordance with the qualitative analyses. As the microscopically observed opacification morphology may already suggest, the highest levels of straylight were observed in the Euromaxx IOL. Compared to the cataract lens, the straylight of the Euromaxx IOL was approximately 8.8-fold higher, while the LS-312 MF30 IOL was 2.4-fold higher. This indicates the serious impact of calcification on the patient’s quality of vision, the calcified IOLs having a more debilitating effect than the cataract they were intended to treat.

In several laboratory studies our research group has shown that the presence of glistenings may degrade the optical performance of the IOLs, affecting the central image quality in patients [16,17,18,19]. Łabuz et al. noted especially that the glistenings may lead to a significant increase in the straylight levels [19].

In our study, we used a method we have successfully used in the past [18] to induce glistenings in the hydrophobic acrylic PC-60AD IOL. We then compared its forward light scatter effects to those of the calcified IOL-explants. The qualitative assessment using the imaging technique revealed that the glistenings-IOL generated less light scatter than the two calcified IOL-explants. While its measured straylight parameter was markedly less than those of the calcified IOLs, it was still approximately 2-fold higher straylight than a 70-year-old lens, implying the clinical relevance of the glistenings in vivo. As size, density, and affected area of the glistenings have been shown to influence the magnitude of light scattering effects [34,35], future studies comparing the forward light scatter pattern of various IOLs with different glistenings’ morphology may be insightful in determining a correlation.

To summarize, the veil of light resulting from the increased light scattering in the opacified IOLs could be visualized off the optical axis using the proposed ray propagation imaging technique. Further research is needed for a quantitative differentiation of the light scattering effects caused by IOLs with different opacification patterns. Both the calcified IOL-explants as well as the glistenings-IOL showed higher straylight levels than those of a clear control IOL. Such high straylight values are indicative of the increased sensitivity to glare and poor optical quality that these IOLs would cause in vivo.

## Data Availability

All data generated or analyzed during this study are included in this published article.
